# A BLE based turnkey indoor positioning system for mobility assessment in aging-in-place settings

**DOI:** 10.1371/journal.pdig.0000774

**Published:** 2025-04-17

**Authors:** Haixin Wang, Guha Ganesh, Michael Zon, Oishee Ghosh, Henry Siu, Qiyin Fang

**Affiliations:** 1 School of Biomedical Engineering, McMaster University, Hamilton, Ontario, Canada; 2 Department of Family Medicine, McMaster University, Hamilton, Ontario, Canada; 3 Department of Engineering Physics, McMaster University, Hamilton, Ontario, Canada; Iran University of Medical Sciences, ISLAMIC REPUBLIC OF IRAN

## Abstract

Indoor positioning systems (IPS) can be used to measure mobility at home, which is an important indicator for health and wellbeing. In this work, we designed and developed a Bluetooth Low Energy (BLE) based IPS that identifies individual users; does not require floorplans; and allows the end-users to perform on-site install/setup. Additionally, a dynamic calibration process is implemented to learn room boundaries based on the distribution of the BLE signal strength. The functionality and performance of IPS system were validated in two residential home settings. Raw and filtered relative signal strength indicators (RSSI) and variability of RSSI were measured during testing. Room detection was determined by comparing a user input location (ground truth) with the IPS detected location for over 300 positions. The IPS produced a 96% accuracy of correctly detecting room location when using RSSI and the additional motion sensors. The use of PIR motion and ultrasonic sensors information provided improved validity when compared with existing indoor positioning systems. The ease of use and modular design of this IPS makes it a good choice for implementation in larger scale smart healthcare monitoring systems.

## Author summary

Mobility is a critical indicator of health status, particularly for older adults with co-morbidities. Accurate, quantitative, and long-term measurements of in-home mobility provide valuable data to the healthcare team for diagnosis and interventions. Existing methods to measure mobility at home have poor compliances and/or are difficult to scale up to large number of users living in community dwellings. In this project, we designed and developed a smart indoor-positioning-system (IPS) technology that allows accurate measurements of in-home mobility, while remains low cost to implement and operate. The turn-key design of the IPS technology can be installed, set up, and calibrated by the end users (e.g., older adults and/or their caregivers) themselves. The system itself is low cost, and its operation requires minimal interactions with the old adult end users. These features significantly alleviated the burdens to the end users, which are key barriers to compliance and effectiveness of such technologies, making it feasible to scale up to large number of users in the community.

## 1. Introduction

The knowledge of a user’s position along with external data parameters like environmental sensor data or vital signatures enable the development of healthcare monitoring applications. In addition, storing the user’s location changes over longer periods of time will provide useful information that relates to behavior analysis and activity monitoring [1]. The Global Positioning System (GPS) is currently the dominate positioning technology, which has been embedded in transportation, mapping, and guidance systems everywhere. For applications predominately indoors, however, GPS is of limited usage due to the difficulty in communicating with GPS satellites as well as the increased requirement for positioning precision. Indoor environments propose a great challenge when it comes to position tracking because of the obstacles and interferences to wireless electromagnetic signals from the building structure. A system that can successfully overcome these challenges will prove to be extremely beneficial for many reasons. Indoor position tracking opens a gateway to several unique applications. A simple example would be automating light fixtures based on presence of certain individuals or devices, which would classify as an IoT application. The knowledge of room detection can be used to analyze room transition patterns and potentially apply wayfinding applications like the one used in a museum [2], shopping malls [3], and airports [4]. It is important to set a fully functioning foundation for an Indoor tracking system because it would serve as the backbone to a plethora of application ideas. The technical, medical, and general applications provide immense benefits and integrating them with a powerful indoor positioning system at its core is our goal.

Indoor tracking has a significant impact when implemented in a clinical setting, it opens several approaches to health monitoring intervention platforms when combining time and location data with measured health parameters. The use of this data would be critical in the development of real time context aware healthcare monitoring applications. For example, the use of indoor tracking would be beneficial for Alzheimer’s and Dementia patients who have a history of getting lost by wandering away from their home. Additionally, the IPS would provide caregivers in long term care facilities a method of monitoring multiple patients efficiently. Caregiver burnout is a serious concern, technology that aims to assist caregivers can have a positive impact for the safety of both caregivers and older adults [5]. This project is working to create a technology that is shaped by the insights of older adults and their goals/needs for independence, in addition to providing support and respite for caregivers.

Position tracking is commonly performed by analyzing signal properties of communication protocols to identify a user/ device’s location. An early 2021 systematic review by Pascacio et al covered the various communication protocols used to develop existing IPS technologies and outlined their similarities and differences [6]. Currently, common IPSs determine location using either Bluetooth [7], WIFI [8] or RFID [9] communication protocols. WIFI is often used as a preferred indoor tracking method because of its speed and integration. However, using WIFI to perform indoor tracking requires extensive battery power usage on tracking devices, which limits the time a user can be tracked. WIFI based systems are ideal for indoor tracking in large indoor spaces like hospitals or industrial buildings. H.-P. Bernhard et al propose the development of an WIFI based presence detection system for an automotive assembly factory [10]. Their system would track the location of assembled cars moving from various testing locations and the location of their corresponding parts that are either added/removed. RFID is like WIFI with high fluctuations in signal strength and a limited measurable distance, however it has a lower power consumption. RFID is harder to implement because most commercial wearable devices like smartwatches and cellphones have BLE and WIFI integrations instead [11]. Additionally, RFID signal strength has a lower detection distance when compared to BLE signals. Bluetooth Low Energy (BLE) works within a 30m radius and has multiple parameters that can be assessed for location tracking applications. Some of these properties include, relative signal strength indicator (RSSI), Angle of Arrival/Angle of Departure (AOA, AOD), and TX power [12]. Bluetooth based IPSs are optimized to determine position at almost the centimetre level which makes them ideal for indoor tracking applications.

The two main types of IPSs are proximity/presence-based vs coordinate (x, y)-based. Mokhtari et al uses BLE tags and a proximity-based approach to perform room level detection and activity monitoring [13]. Their research concluded that proximity-based systems struggle with accurate detection during longer recording periods because of data saturation with several room transitions. Noertjahyana et al. developed a similar system except using the trilateration approach [14]. The IPS developed here focuses on room level detection (proximity) with the use of RSSI and motion/ultrasonic sensor feedback. Their IPS uses a combination of BLE and sensor data to confirm whether the tracked individual is present in a room. The main advantage here is that a proximity-based detection system can be implemented at any point of interest, without prior knowledge of room topography. In contrast, the trilateration approach is dependent on processing power and improves as the number of beacons relaying information increases. Trilateration uses several matrices of calculated RSSI based distance values to coordinate an exact x, y position within a known indoor location setting.

Smart devices should prove beneficial to the user, be comfortable, non-intrusive, and easy to integrate. Successful smart home devices must be able to dynamically adapt to any home environment and still function at the highest efficiency possible. Many existing IPS require knowledge of detailed building topography for successful implementation and functionality. This means that these systems would require extensive work on their setup and calibration process. Signal integrity is the most important aspect of all data acquisition systems. The BLE communication protocol is constantly evolving and has progressed to version 5.3 [15], offering enhanced capabilities in range, speed, and power efficacy. However, BLE 4.0 provides proven reliability and extensive documentation, making it an optimal choice for scenarios where these attributes are prioritized over the latest advancements. Its stability and compatibility with existing systems ensure seamless integration and maintainability, affirming its continued relevance and effectiveness for our needs. In this work, an IPS is developed that requires no prior knowledge of room topography with a minimal setup and configuration process. However, it still maintains a high degree of precision and accuracy using BLE signal analysis and environmental sensor data. The system uses compact wall adapter beacon enclosures in conjunction with BLE tags as tracking devices. The system prioritizes being extremely easy to integrate and configure while retaining an extremely high degree of indoor presence detection accuracy.

One fundamental aspect of this system is its adaptability within various indoor environments. Previous literature on indoor tracking proves that in an indoor space, the presence of furniture and wall introduce high levels of signal loss and are the source of RSSI fluctuation [16–17]. Therefore, there is a need for an IPS that can successfully adapt within any indoor environment regardless of fluctuations. Developing an IPS that does not have to be preprogrammed based off building topography and room layout is another reason why adaptability is vital. This system would avoid several challenges created by currently developed IPSs. Some of these challenges include the need for a floorplan to design an optimal setup configuration prior to installation. Additionally, this system would avoid the need for any professional installation procedures such as wall mounting beacons in hard-to-reach locations, which is commonly required. A system like this would make integration within large buildings like hospitals or retirement homes significantly easier and still maintain a high degree of efficiency.

## 2. System design

The IPS we developed leverages BLE technology to accurately measure user mobility within indoor environments. The system is composed of several key components, including ambient BLE beacon modules, wearable BLE tags, and a central hub for data processing and storage. The IPS operates by detecting the signal strength from wearable BLE tags, such as iTags or smartwatches, using multiple BLE beacon modules placed throughout the environment. These beacons relay the sensor data to a central hub module, which then processes the information to determine the user’s position.

Our analysis is grounded in two key theories: signal propagation in indoor environments and context-aware computing. Signal propagation theory helps explain how wireless signals, such as RSSI, behave in indoor environments, affected by obstacles, interference, and distance. This theory underscores the challenges of accurate positioning in complex spaces, which we addressed by applying an exponential filter to reduce signal fluctuation. Context-aware computing theory is central to understanding how location data, combined with environmental and activity information, can provide insights into user behavior. While this framework is effective in mobility tracking, its limitations include the difficulty of integrating diverse data sources and the reliance on precise contextual data. By critically engaging with these theories, we situate our work within the broader literature on indoor positioning and healthcare monitoring, addressing gaps such as adaptive systems that require minimal setup and deliver high accuracy in dynamic environments.

**Fig 1:**
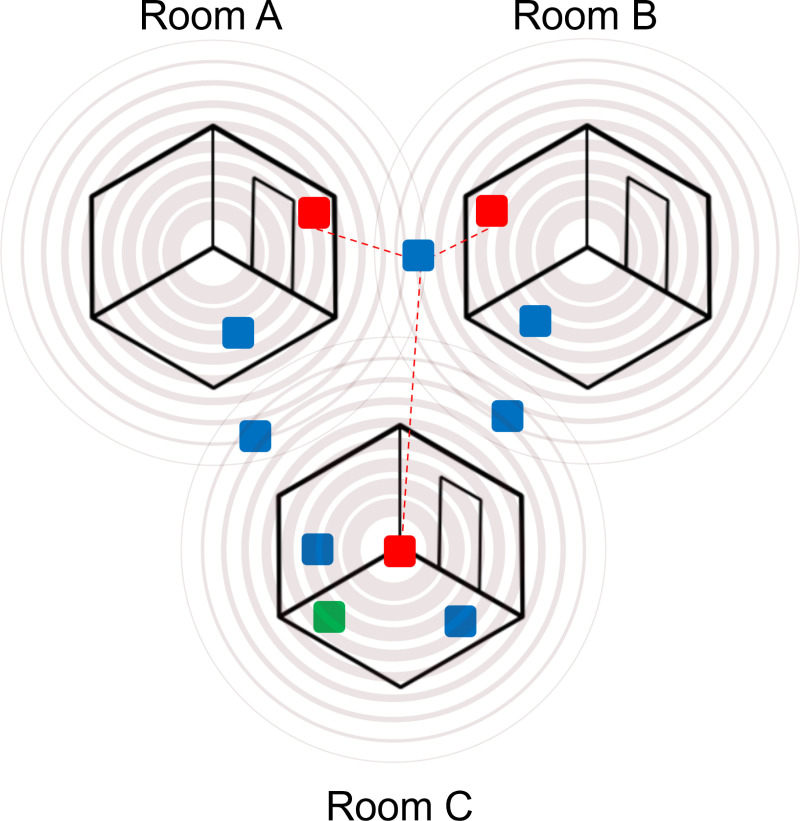
IPS System level design diagram, Red: beacons in each room including an ESP32 and sensors, Green: a data acquisition hub containing a Raspberry Pi and an ESP32 microcontroller, Blue: wearable BLE tags (iTag or smartwatches). Different rooms can be functional areas in a home such as: kitchen, hallway, bathrooms, bedrooms, living room, study/office etc.

Fig 1 illustrates the core functionality of the IPS, highlighting the major components and their interactions. The system’s ability to estimate positions without requiring detailed floor plans is achieved through two primary techniques: calibration-based room identification and trilateration.

During the initial calibration phase, the system establishes baseline RSSI values for each room by recording signal strengths at known positions (ground truths). By maintaining a running average of these RSSI values, the IPS dynamically adapts to recognize the unique signal strength patterns of each room. This self-adjusting mapping allows the system to identify the current room based on real-time RSSI readings, effectively eliminating the need for physical floor plans. This method ensures that the system can function in various environments by learning and adapting to the specific characteristics of each space. For more granular position estimation, the IPS employs trilateration, which involves measuring the signal strength from at least three different beacons to determine the user’s location. Each beacon provides an RSSI value that correlates with a distance radius from the beacon. The intersection of these radii allows the system to triangulate the exact position of the user within the environment. This technique is particularly useful in larger or more complex spaces where multiple functional areas exist, ensuring accurate tracking even in challenging settings. The combination of calibration-based room identification and trilateration enables the IPS to operate effectively in a wide range of indoor environments, from small residential spaces to large, complex buildings. The system’s modular design facilitates easy integration and scalability, making it suitable for deployment in diverse settings such as hospitals, retirement homes, and smart home environments.

All sensor data collected by the beacon modules is transmitted to a central hub, typically a Raspberry Pi, which processes and stores the data locally. This approach ensures reliable data handling and supports real-time monitoring and analysis.

To validate the system, we conducted a comprehensive review of recent studies. Pascacio et al. (2021) highlighted BLE’s cost-effectiveness and precision in room-level detection, aligning with our system’s strengths [6]. Mokhtari et al. (2018) confirmed BLE’s practicality for activity tracking with minimal setup [13], which supports our methodology. However, Jondhale et al. (2016) identified challenges in using RSSI for precise localization, which we mitigated through our application of an exponential filter [18]. These studies demonstrate the feasibility of BLE for healthcare monitoring while emphasizing areas for further optimization, such as improving calibration and addressing environmental variability. Our IPS demonstrates significant advancements in indoor positioning by combining adaptive calibration techniques and precise trilateration. The system’s ability to function without detailed floor plans and its ease of integration make it a valuable tool for enhancing health and wellbeing monitoring in various indoor environments. This innovative approach not only addresses the limitations of existing IPS technologies but also paves the way for future developments in digital health.

### 2.1 Hardware and electrical

**Fig 2:**
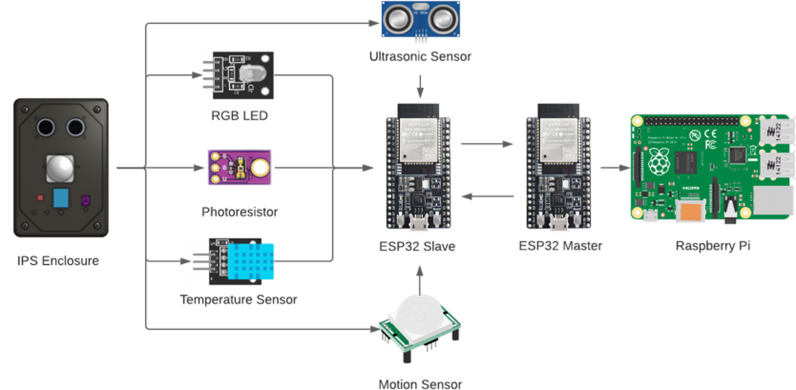
Hardware Flow Diagram, Red: Beacon Module Component Composition, Green: Hub Module Component Composition. The red outline corresponds to multiple BLE beacon modules connected to outlets in the tracked areas. The green outline consists of one hub module that contains a microcontroller and Raspberry Pi connected via micro-USB. This hardware setup ensures all data arrives at a central location and is processed on a separate device.

The overall hardware system consists of three major components: sensors, microcontrollers and BLE tracking devices. The sensors and microcontrollers are physically connected to each other whereas the BLE devices can be standalone and communicate with each microcontroller using Bluetooth signal communication. Each beacon consists of four sensors (Ultrasonic, PIR Motion, Ambient Light, and Temperature) connected to a single microcontroller (ESP32). The electronics in the beacons are powered by 5VDC from an AC-DC adapter integrated on the enclosure, which can be directly plugged into a standard residential power outlet. Fig 2 illustrates the Hub – Multiple Beacon approach used.

**Fig 3:**
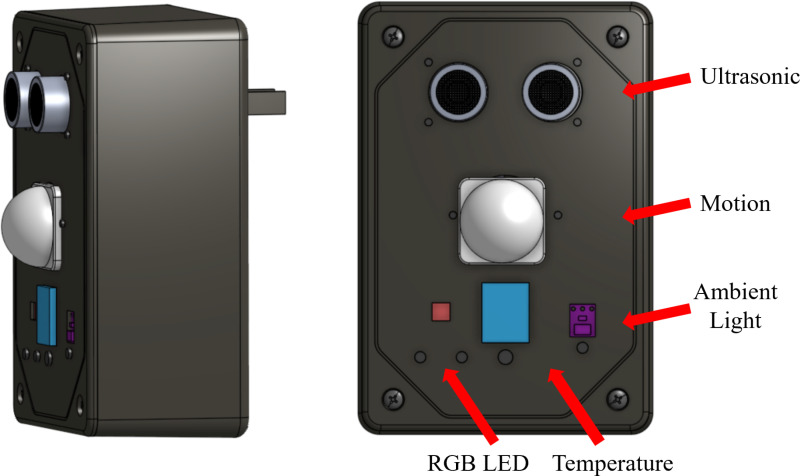
Beacon Module Enclosure with Labelled External Sensors.

The beacon module is a custom designed electronic device that encompasses sensor measurement, BLE signals and Wireless communication using a microcontroller to process these data points. Each module uses the ESP32-Devkit-C as its microcontroller unit. The ESP32 is the core of the IPS as it handles all BLE and sensor data communications. The electrical and mechanical components are labelled as the IPS enclosure in Fig 3. Inside each beacon module is a custom-built PCB that connects all the sensors and microcontroller and eliminates the need for perf boards or breadboard-based connections.

The enclosure is a customized 3D printed case made of Polylactic Acid (PLA). It consists of a lid that contains mounting options for sensors and a base that encloses the AC to DC power adapter and customized ESP32 print-circuit-board (PCB) shield. The PCB was designed using Autodesk Eagle and contains two layers with all sensor connections and microcontroller mounting on the top layer.

### 2.2 Software and data architecture

**Fig 4:**
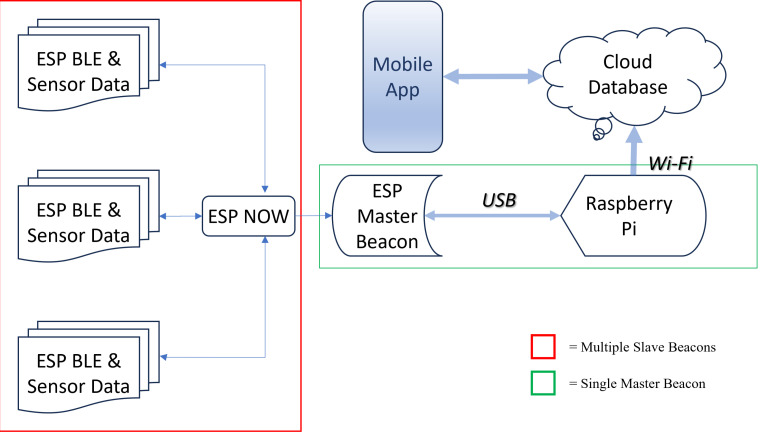
Software and Data Transfer Flow Chart, Red: Beacon module sensor and BLE data communication pathway from rooms to the hub module, Green: Hub module post processing data management, allocation, and calibration process pathway. A customized mobile app was developed for real time calibration and experimental validation purposes.

The software component of the IPS is split amongst the different devices being used. The microcontroller uses the C++ programming language to perform BLE signal acquisition and filtering along with all the sensor data collection. The Raspberry Pi 4 uses Python to perform data parsing methods and wireless transfer of BLE and sensor data to a Google Firestore cloud database. The data acquisition process and relationship between the components within the IPS is shown in Fig 4.

Four types of communication protocols are used in this IPS: ESP-NOW, WIFI, BLE and USB-UART Serial. ESP-NOW is a 2.4GHz frequency-based communication protocol developed by Espressif. It uses a peer-to-peer communication approach, which is why we chose to design a Hub Module – Multiple Beacon Module approach. It is used here to send BLE signal strength and sensor data between microcontrollers. The beacon modules receive advertising packets from known tags using BLE communication and sensor data from the physical sensors on the beacon. The signal strength from these devices is stored momentarily on the microcontrollers and are sent along with sensor data to the hub module using ESP-NOW communication. The hub module will continuously receive data flow from several beacons within the indoor environment.

When any data is received by the hub microcontroller, it will relay this data to a hub via serial communication. Physically, the hub will contain both a microcontroller (ESP32) and a microcomputer (Raspberry Pi) that are connected serially via micro-USB. All transferred data is saved locally on the Raspberry Pi and is periodically sent to Google Cloud Firestore over the Internet.

After installation at a user’s residence, it will go through a user administrated calibration process, which will generate a dataset containing ground truth locations and associated signal strength. A customized algorithm was developed to use this dataset to calculate real time location during regular operation.

### 2.4 In home setup and operation

Ethical considerations were integral to our study. Participants were fully informed about the nature of the data being collected and its intended use. Consent was obtained with assurances of voluntary participation and the ability to withdraw at any time. Data anonymity and security were maintained rigorously, with all collected data stored in a non-identifiable format to prevent misuse. Participants retained ownership of their data, and we worked closely with privacy experts to establish robust security measures. These efforts ensured that participants’ autonomy and confidentiality were protected throughout the study.

To use the IPS in a residence, it will be first installed and calibrated using a process that can be administrated by the user without the need for a floor plan. This is one of the key features of this IPS design, which makes it practical and feasible to be used in a common household. First, the user will install each pre-labeled beacon to an outlet in a specific room/functional area (e.g., bedroom, livingroom, kitchen, etc.) Next, the user (with the tag) is asked to enter each functional areas while recording the time while in that area. This calibration dataset will be used to train the localization algorithm.

For example, to calibrate a beacon placed in the bedroom, the user would walk around the perimeter of the room for a period of 20 seconds. During this period, the system dynamically records signal strength values from all surrounding beacons and determines a range of RSSI fluctuation patterns within the room itself and its sublocations. Both the signal strength data and the starting-ending time in that room will be recorded as the calibration dataset for the bedroom.

Before setting up the IPS in a participant’s home, we conduct an initial survey to understand their specific living situation, such as the number of rooms to be tracked. This step is crucial for customizing the system based on the participant’s privacy concerns and habitual movement patterns in different rooms or functional areas. The setup process is designed for ease of use; it involves plugging the hub into a power outlet and connecting it to the internet via an Ethernet cable. This internet connection is optional based on the participant’s comfort level regarding privacy. Notably, no data is stored on a cloud server; the data stored in Firebase is temporary and is solely used for real-time monitoring of beacon values to ensure the system’s smooth remote operation.

After this initial step, each beacon is plugged into an outlet in the designated functional areas or rooms to be monitored. We advise avoid positioning two beacons on opposite sides of the same wall to maximize spatial coverage. Participants are then asked to wear a smartwatch or a Bluetooth tag, except during charging periods. Ideally, the device should be charged nightly, though its battery can last 3-5 days.

Data collection is flexible, allowing manual retrieval from the Raspberry Pi or over-the-air transfer if connected to the internet. Our system supports both initial and continuous self-calibration. For initial calibration, the participant wearing the watch or tag is asked to spend 3-5 minutes in each monitored room, establishing a baseline for our machine learning model for room identification. This calibration can be adjusted over time to accommodate changes in the floor plan. Additionally, the infrared and ultrasonic motion sensors in each beacon provide a cross-validation mechanism for presence detection in each room, enhancing the system’s accuracy and reliability.

## 3. Performance evaluation and results

### 3.1 Experimental setup

The functionality and performance of the IPS were evaluated in two residential houses with simulated activities. Testing parameters were documented and tabulated prior to conducting each individual test in Table 1. The two houses where experiments were conducted are in suburban residential neighborhoods in the City of Mississauga and Hamilton (McMaster Smart Home for Aging-in-Place (SHAPE) facility), both of which are in the Greater Toronto Metropolitan Area (GTA). Conducting the same experiments in two different locations allowed for analysis of environmental changes and improved validity in the system’s functionality. The suburban residential neighborhood setting provides a typical wireless signal environment, e.g., WIFI, Bluetooth, cellular networks, etc. Both houses are typical single-family dwellings with multiple stores (two floors plus basement). The house in Mississauga (House 1) contains typical residential household electrical outlet settings (one per wall). The SHAPE facility (House 2) is a house with special electrical wiring systems that has multiple outlets per wall.

**Table 1:** Experimental test log and parameters.

Test Subject	Type of Test	Tested Device	Height (m)	Device Position	Device Location	Orientation Relative to Beacon
#1	RSSI Stability (1m)	Tag	1.54	Around Neck on Pendant	House 1 - Family Room	Directly Facing Beacon
#1	RSSI Stability (2.5m)	Tag	1.54	Around Neck on Pendant	House 1 - Family Room	Directly Facing Beacon
#2	RSSI Stability (1m)	Watch	0.48	On Right Wrist	House 1 - Family Room	Directly Facing Beacon
#2	RSSI Stability (2.5m)	Watch	0.48	On Right Wrist	House 1 - Family Room	Directly Facing Beacon
N/A	RSSI Stability (1m)	Watch	0.65	Flat on Desk	House 2 - Basement	Facing Upwards
N/A	RSSI Stability (1m)	Tag	0.65	Flat on Desk	House 2 - Basement	Facing Upwards
#1	Room Detection - RSSI	Watch	0.65–1.27	Wrist	House 1	N/A
#2	Room Detection - RSSI	Tag	0.65–1.27	Wrist	House 1	N/A
#1	Room Detection - RSSI	Watch	0.65–1.27	Wrist	House 2	N/A
#2	Room Detection - RSSI	Tag	0.65–1.27	Wrist	House 2	N/A
#1	Location vs Ground Truth	Watch	0.65–1.27	Wrist	House 2	N/A
#2	Location vs Ground Truth	Watch	0.43–1.06	Wrist	House 2	N/A
#1	Location vs Ground Truth	Tag	0.65–1.27	Wrist	House 1	N/A
#2	Location vs Ground Truth	Tag	0.43–1.06	Wrist	House 1	N/A

The experiments required human test subjects are labelled as subject 1 and 2 respectively. Additionally, only two types of devices were used for the tests, a smart watch (Amazfit S2, Mobovi) and a compact Bluetooth tag (iTag, Universal Ascent Holdings Limited). Certain tests do not have their orientation labelled because the device is constantly moving and does not remain in one fixed orientation relative to the beacon.

### 3.2 RSSI fluctuation and filtering

Initial testing was conducted to determine static fluctuation in RSSI when a smartwatch and tag are in a still position. Stability tests were performed at fixed distances of 1, 2.5, 5 and 10 m from a single beacon in two different test environments. Tests were performed in an interval of 100 seconds with a test subject standing at a fixed position with the device of choice. During this interval, the relative signal strength indicator (RSSI) is plotted. The objective of this experiment is to observe the effects of RF interference on BLE signal strength and determine how effective filtering is on these noisy RSSI signals. Additionally, the key differences observed between distance and signal strength will prove that the use of signal strength analysis is an effective method to determine location or presence. Equation (1) will be used to calculate the distance based off measured RSSI values for both raw and filtered data. The measured power would be the estimated RSSI at 1m distance from the beacon. This value varies depending on the beacon used to measure RSSI. N is an environmental factor that ranges between 2-4 and is determined after correlating the calculated distances with fixed ground truth distances. The RSSI value is the measured signal strength. Using this equation, the resulting distance vs time graphs will be plotted for further analysis.


$$Distance=10^{\frac{\left(MeasuredPower-RSSI\right)}{10\times N}}$$ (1)

The filtering performed in this paper consists of a simple exponential filter applied on raw RSSI values in real time. An exponential filter works using a recursive algorithm and prioritizes the previously filtered value along with a filter weight to accurately determine the newly filtered value y_n_ as shown in equation (2). The variable x_n_ holds the measured raw RSSI value and the variable y_n-1_ holds the previously calculated filtered value. When analyzing the filter’s performance, it is important to consider the weight factor “w”. Several researchers use similar filters that operate using a weight factor or similar constants like the Kalman filter and Particle Filter [19–20].


$$y_{n}=w\times x_{n}+\left(1-w\right)\times y_{n-1}$$ (2)

Throughout experimental analysis of RSSI fluctuation data, various filter weights were used, and newly filtered datasets were obtained. However, for the functional IPS, an optimal filter weight was desired. To accurately determine what value of “w” is required further analysis of the exponential filtering on RSSI values was required. To determine this value, the root mean square (RMS) of filtered RSSI fluctuation datasets were calculated and plotted. Each dataset contained 100 RSSI values that were filtered using weight factors that varied from 0-100%.

**Fig 5:**
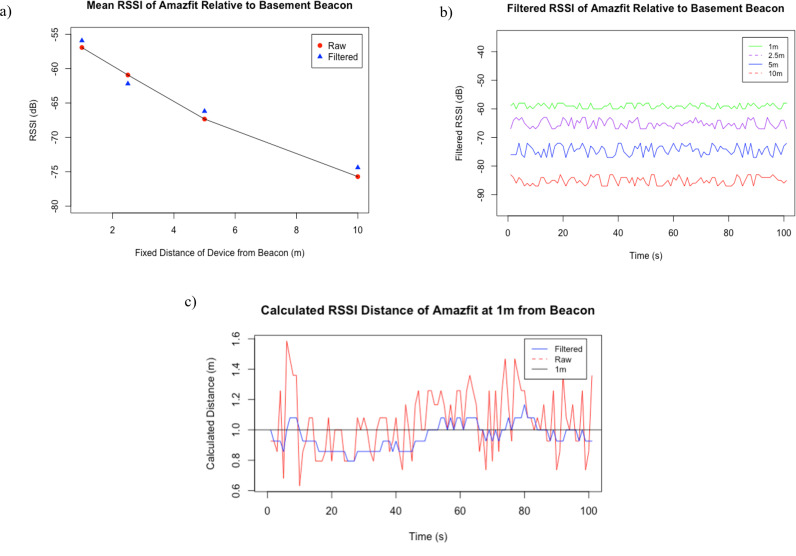
RSSI value of the Smartwatch (Amazfit): top left (a): Mean Measured RSSI at Fixed Distances (1, 2.5, 5 and 10m) in the Smart Home Basement, top right (b): Filtered RSSI at Fixed Distances (1, 2.5, 5 and 10 m) in the Smart Home Basement, bottom (c): Differential RSSI value (calculated distance) fluctuation when a tag is placed statically at 1m distance from a beacon.

Fig 5a displays an RSSI vs Distance graph from average RSSI measurements taken during interval tests in the McMaster Smart Home basement. The figure shows raw and filtered RSSI levels, and their calculated distances based off Equation 3.1.

The raw and filtered RSSI values of the smartwatch at distances (1, 2.5, 5 and 10 meters) are graphed and illustrated in Fig 5c. Graphed raw RSSI values show rapid changes at every measured distance while maintaining a reasonably distinguishable range. Graphed filtered RSSI values show smaller changes and have clearly distinguishable ranges. The observed RSSI ranges are approximately -50 to -60 at 1m, -55 to -65 at 2.5m, -65 to -75 at 5m and -75+ at 10m.

In addition to the plotted raw and filtered RSSI, the measured datasets were analyzed to determine standard deviation, mean RSSI and variance. The calculated standard deviation values of the smartwatch during the 10 m-test in the Residential Home was 1.68 – Filtered. In the McMaster Smart Home, the 10m test results was 1.90 – Filtered. Testing was performed using an additional device known as an iTag for comparison between smartwatch and BLE tag RSSI values. The calculated standard deviation values of the tag during the 10m test in the Residential Home were 2.57 - Raw and 1.56 – Filtered. In the McMaster Smart Home, the 10 m test results were 2.21 – Raw and 1.74 – Filtered.

**Fig 6:**
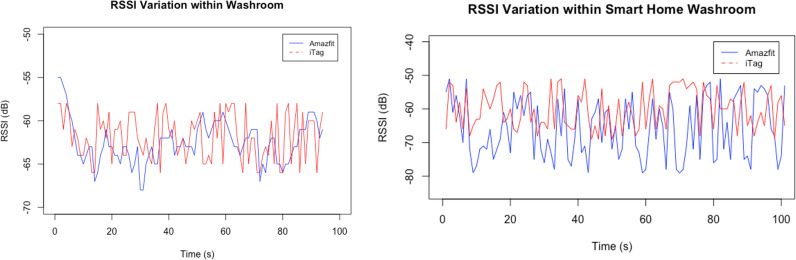
RSS value filtering: left (a): Exponential Filtered Sin(x) Function at Varying Weight Factors, right (b): RMS value as a function of Weight Factor for the RSSI Stability Dataset (1m).

Testing of the generic sinusoidal function revealed that the phase shift is not affected by the filter, however the amplitude is, as shown in Fig 6a. The sin(x) function remains the same at a weight factor of 1 (100%) and gradually smoothens as weight is decreased. It is evident that at lower weights (w = 0.5 and 0.2), the filtered function responds slowly to changes that are evident in the original signal (w = 1). Using the filtered Sin(x) function’s graphed response, a similar process was applied to a singular dataset from the RSSI fluctuation tests to produce Fig 6b. The RMS curve follows an exponential growth between 0-20 weight % and then steadily increases.

### 3.3 Room RSSI variation

To ensure room detection would be as accurate as possible, RSSI fluctuations were measured in various rooms within the McMaster Smart Home and Residential Home. For this experiment, a user would be wearing a smartwatch on their wrist or a Bluetooth tag pendant around their neck while they walk around a room for a period of 100 seconds. Tests were conducted in 4 rooms at both testing locations. The purpose of this experiment is to record RSSI variations through rooms of various sizes.

**Fig 7:**
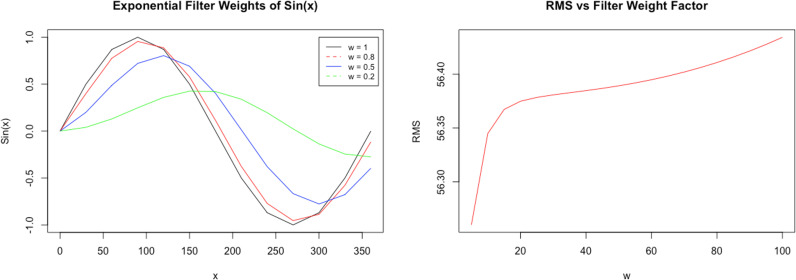
Measured RSSI value over time in: left (a) the Residential Home Washroom, right (b): the McMaster Smart Home Washroom.

Measured RSSI changes from both the smartwatch tag while moving within a single room were measured and graphed. Graphed RSSI in the washroom of the residential and smart home show a similar range for both devices as shown in Fig 7a, b. Bedrooms and workspaces produced higher RSSI variation where RSSI reached a minimum value -88 dB and maximum of -55 dB. In the case of the washroom RSSI data, the maximum value recorded was -46 dB and the minimum was – 64 dB.

Further analyzed properties like standard deviation, maximum and minimum RSSI were calculated displayed in Table 2. A maximum standard deviation of 5.68 dB was calculated from the smartwatch in the residential home bedroom. A maximum standard deviation of 5.61 dB was calculated from the tag in the residential home office room. In the McMaster smart home, similar maximum standard deviation values were calculated and are greater than 5 as well.

**Table 2:** RSSI Signal Properties of the smart watch and Tag.

Room	Tag (dB)			Smart watch (dB)		
	Mean	Max	Min	Mean	Max	Min
House 1 – Residential Home						
Bedroom	−76.45 ±4.67	−56.00	−84.00	−73.66 ±5.68	−55.00	−80.00
Washroom	−59.65 ±2.54	−48.00	−61.00	−56.76 ±2.89	−52.00	−64.00
Office	−74.43 ±5.61	−59.00	−88.00	−77.65 ±5.43	−59.00	−86.00
Bedroom 2	−54.32 ±2.43	−46.00	−58.00	−56.51 ±2.87	−50.00	−61.00
House 2 - SHAPE Facility						
Bedroom	−66.84 ±5.23	−57.00	−82.00	−68.40 ±5.32	−51.00	−78.00
Washroom	−56.67 ±2.45	−52.00	−61.00	−53.45 ±2.13	−46.00	−59.00
Office	−71.21 ±5.12	−61.00	−84.00	−65.64 ±5.17	−54.00	−74.00
Kitchen	−54.89 ±2.77	−51.00	−60.00	−58.63 ±3.25	−49.00	−60.00

Note: RSSI value of a specific tag measured by the beacon in the specific room.

Rooms will produce varying RSSI values when a user is moving around the beacon’s relative location. Analyzing these values allows us to determine a range and calculate the standard deviation of RSSI for each room. The variation proved to be significantly lower in smaller rooms when compared to larger rooms as depicted in Table 2. To accurately detect if a user is within that room multiple factors must be considered along with the RSSI values. This test proved that a calibration setup is required to accurately determine room presence based of a dynamically measured threshold. Room size variation made detecting indoor position at the sub room level difficult for the smaller room sizes. Potential ways to compensate for this would be to enhance the calibration algorithm to react differently on RSSI changes in smaller rooms compared to larger ones.

### 3.4 Location vs. ground truth

A method of validating whether a user is in the detected room was required to successfully assess the quality and efficiency of the IPS. This validation experiment was performed using a custom designed mobile application that seeks user input on a user’s current room location. The mobile app required a user to enter a room, wait 10 seconds and validate the room they are currently in (Ground Truth). This selection is then compared with the calculated location that the IPS determined based off signal strength (Location). 150 room selections were completed by two separate test subjects as they traversed between either 4 or 5 rooms depending on the test location.

Mobile app entries determined locations were compared, the detection results are displayed in Table 3. The IPS achieved a calculated percentage accuracy of 96.7% in the residential home and 95.3% in the smart home.

**Table 3:** User input location vs ground truth location test results.

	Subject 1	Subject 2	Total
House 1 – Residential Home (4 Rooms)			
Number of Tests	150	150	300
Total Correct Location Matches	146	144	290
Incorrect Location Matches	4	6	10
% Accuracy	97.33	96.00	96.67
House 2 - SHAPE Facility (5 Rooms)			
Number of Tests	150	150	300
Total Correct Location Matches	144	142	286
Incorrect Location Matches	6	8	14
% Accuracy	96.00	94.67	95.33

### 3.5 Validation of sensor based room detection

The addition of sensors along with BLE signal strength analysis provides meaningful data that can be analyzed in real time or post processed. The IPS is equipped with a PIR motion sensor (HC-SR501), Ultrasonic Range Finder (HC-SR04), Ambient Light sensor (TEMT6000) and a DHT-11 temperature sensor. The ultrasonic and PIR motion sensors were primarily used for motion detection with temperature and ambient light used for context awareness applications. Following a similar process as test #3 (Location vs Ground Truth), the motion and ultrasonic distance measurement thresholds were compared with a user input location. For example, when walking into “room 1” the expected sensor output from room 1’s beacon should detect presence via the motion sensor and fall within the calibrated threshold for the ultrasonic sensor. The mobile app required a user to enter a room, wait 10 seconds and validate the room they are currently in (Ground Truth). This selection is then compared with the calculated location that the IPS determined based off the motion sensor and ultrasonic sensor outputs. 150 room selections were completed by two separate test subjects as they traversed between either 4 or 5 rooms depending on the test location.

User indicated motion entries (Ground Truth) and recorded sensor values were compared, and the detection results are displayed in Table 4. The motion sensor achieved a total 93% accuracy, defined by the percentage of correctly detected instances. The ultrasonic sensor at a 200 cm threshold produced a lower accuracy of 78.67%. Temperature and ambient light sensors were tested for functionality and successfully relayed their measured values in real time after a beacon is connected.

**Table 4:** Results of motion and ultrasonic range detection testing1.

	Subject 1	Subject 2	Total
Motion Detection Testing			
Number of Tests	150	150	300
Correct Motion Detection (Presence Detected)	141	138	279
Incorrect Motion Detections (Presence Not Detected)	9	12	21
% Accuracy	94.00	92.00	93.00
Ultrasonic Detection Testing (2m Threshold)			
Number of Tests	150	150	300
Correct Ultrasonic Detection (Within Threshold)	126	110	236
Correct Ultrasonic Detection (Not Within Threshold)	24	40	64
% Accuracy	84.00	73.33	78.67

### 3.6 Detection speed (time) during room transitions

Performance testing of the IPS involves determining how fast it can detect room changes and presence. This experiment consisted of a user traversing between two adjacent rooms of similar size while the time difference between timestamped presence detection is compared to determine detection speed, measured by the time needed to detect the tag. The same experiment was repeated for rooms that are at greater distances apart from each other.

Each room transition was performed 15 times for both the smart watch and tag. The average detection time was calculated and reported in Table 5. The results show that the transition time correlates with the physical distance between the two rooms (e.g., “adjacent” versus “far”). Although there are small differences between the measured transition time between the two types of tags, the differences are small enough to be negligible for the purpose of daily activity or mobility measurements.

**Table 5:** Results of room transition time (seconds).

	Smart watch	Tag
Residential Home		
Bedroom to Washroom (Adjacent)	1.56	1.37
Washroom to Prayer Room (Adjacent)	2.54	1.91
Prayer Room to Office (Far)	4.53	5.87
Office to Bedroom (Far)	5.69	7.41
Smart Home		
Washroom to Office (Adjacent)	2.81	2.32
Office to Kitchen (Adjacent)	1.12	1.88
Kitchen to Dining Room (Adjacent)	2.53	3.12
Dining Room to Bedroom (Far)	5.62	6.31

## 4. Discussion

Throughout the development of this IPS several objectives were targeted. Our aim was to develop a system that can measure indoor locations at the room level, accurately determine room transitions, identify traversal pathways of specified BLE devices, correlation of room detection with timestamped sensor data, self-installation with minimal or no house visits, a reasonable cost to develop and secure data collection. The results of our validation tests directly align with several objectives mentioned above and are further outlined in detail below. The physical design of the IPS beacons and hub deal with the objective of self-installation. Beacons are built to be connected directly into wall sockets to eliminate the need for battery replacement or charging. The hub module follows the same process and has one additional connection to either a home router or ethernet port anywhere within the home. This design made the system extremely user-friendly asking for minimal effort from the user during setup and installation. The reasonable cost objective was achieved as the system (assuming 5 beacons/rooms on average) costs approximately $200 to build. To ensure all data remains secure all collected sensor and Bluetooth data remains on the hub module device saved locally. The communication of data between beacons to the hub all operates on a secure 2.4 GHz channel without any need for internet connectivity. The design, testing and validation of this IPS took all these objectives into consideration throughout the entire engineering design process.

In indoor environments, it is evident that the RSSI at a stable position produced high levels of fluctuation due to RF interference within an indoor environment. Obstacles like furniture and metal properties within walls can have a serious impact on the RSSI values [21]. When raw RSSI is left alone there is substantial overlap between values at short distances as shown in the results from Fig 5c. When the exponential filter is applied to the raw values, determining the relative distance of a device is significantly easier as shown in Fig 5b. This decrease proves that filtering the RSSI values provides a significant advantage for the indoor tracking algorithm.

With regards to the main objective of room detection, a clear difference in measured RSSI is evident as distance increases. RSSI strength decreases as the tracked device moves further away from the beacon. Fig 5b and c show a clear difference in the efficiency of using filtered RSSI vs raw RSSI. Filtered RSSI plots produced significantly less deviation making it easier to correlate a distance to determine presence.

The RSSI changes based off the implemented exponential filter significantly improve the signal quality. Results from the fluctuation and room variation tests prove that the filter aids the IPSs ability to accurately detect rooms. From Fig 7b, there is a large spike in the RMS value through the 0-20% weight range. From previous testing, it was noted that larger weight factors significantly increased the processing time and power consumption. Therefore, the optimal weight would be to use 20% as it is the smallest weight that maintains a smooth filter at the fastest possible processing speed.

As a user navigates around a beacon, RSSI values fluctuate across different rooms. By analyzing these variations, it is possible to determine a range and calculate the standard deviation of RSSI for each room. The variation proved to be significantly lower in smaller rooms when compared to larger rooms as depicted in Table 2. To accurately detect if a user is within that room multiple factors must be considered along with the RSSI values. This test proved that a calibration setup is required to accurately determine room presence based of a dynamically measured threshold. Room size variation made detecting indoor position at the sub room level difficult for the smaller room sizes. Potential ways to compensate for this would be to enhance the calibration algorithm to react differently on RSSI changes in smaller rooms compared to larger ones.

Comparison between user input locations and detected locations resulted in a high % accuracy for the overall IPS. A difference of around 1% was observed in the calculated % accuracy between the two test locations. This result supports the IPS’s ability to adapt to new indoor environments [Table 4]. The physical act of transitioning between rooms causes the IPS to receive multiple RSSI signals that are within a similar range to the closest available beacons. The similarity in RSSI range is the cause of the incorrect location detection. To mitigate this problem, filtering of RSSI values can be improved along with the inclusion of a more extensive calibration process during integration. Future experiments will be conducted using more than two test subjects and more than five rooms to detect. Additionally, a greater number of room transitions would be added to the testing procedure. The results of this experiment truly ensured that the system was correctly detecting the presence of an individual traversing within their home. It aligned with the objective of using IPS data to observe room traversal patterns and pathway guidance application development.

During sensor-based room detection, results indicated that the motion sensor serves as an effective backup for validating room detection due to its high accuracy rate. The ultrasonic sensor produced a significantly lower % accuracy, with several false positive room detections. Limitations on ultrasonic threshold distance and angle are the reasons for the lower accuracy. The ultrasonic sensor has a maximum distance measurement of 400 cm directly in front of its detector. Whereas the PIR motion sensor has a 5m hemispherical radius in front of the detector. A drawback to using physical sensors for room detection is the location of the beacons will become limited. The detector heads from the motion and ultrasonic sensors must be facing an open area to accurately detect if a person walks in front of or past it. The beacons are designed to act as wall adapters and throughout most residential and clinical settings outlets are often covered by furniture or equipment.

In terms of room transition detection and detection speed, results showed longer detection times for more distant rooms, which aligns with the increased time required to traverse larger spaces. Commercial indoor positioning systems can determine room location at less than 1 second speeds with prior knowledge of room layout and building topography [22]. Some possible reasons for the longer detection time observed would be the ESP NOW communication delay. BLE data is sent across a different communication channel at a different operating frequency. Additionally, the added filtering process could potentially add a significant delay due to processing incoming and past RSSI data before room detection analysis even begins. Further testing and validation need to be performed to confirm these concerns and determine if these are reasons for the longer speeds. Future work would involve exploring methods to increase the measured detection speed to achieve similar speeds without the need for knowledge of room layout. The detection speed and room transition data aligned with our objective of using IPS data to observe room traversal patterns and pathway guidance application development.

Overall, the development and validation of this IPS have effectively met multiple design objectives, enhancing the capability to detect room-level locations, transitions, and user pathways with considerable precision. Through the integration of an exponential filter, the system effectively reduces RSSI fluctuations caused by environmental factors, thereby improving the accuracy and reliability of distance estimations and room presence detection. The results demonstrate that the IPS can adapt to diverse indoor environments and detect subtle variations in room sizes, which facilitates accurate room detection and pathway guidance. The use of motion sensors as a backup for room detection further validates the robustness of the system. Future improvements will focus on refining RSSI calibration and enhancing sensor integration to decrease detection times and expand the system’s applicability. This comprehensive approach ensures the IPS remains versatile and effective in various indoor settings, making it a valuable tool for real-time location-based services.

## 5. Conclusion

Longitudinal tracking of relevant daily activities can provide useful information for better understand and management of older adults’ health [23–24] and general wellbeing [25–26] applications. Indoor position tracking continues to improve in methodology and implementation as technology advances, employing diverse methods such as WiFi, BLE, RFID, and meticulously mapped topographies to achieve precise device tracking, often down to the centimetre level. However, the system design in this paper performs location tracking at the room level without any preprogramming requirements using a dynamic calibration process and filtered BLE RSSI signal strength analysis. Additionally, the integration of motion and ultrasonic sensors further enhances the system’s ability to validate its location tracking autonomously, adding a layer of reliability and accuracy.

In validating this system, a series of experimental procedures were undertaken to assess its capability in accurately determining the locations of BLE tags and smartwatches. Key aspects such as RSSI quality, variations, the correlation between calculated locations and ground truth, and sensor-based room detection were meticulously tested. The system demonstrated robust performance across these parameters, offering a promising solution for tracking human subjects equipped with wearable technologies like smartwatches or pendant tags.

Moreover, the flexibility of this system extends beyond human tracking; it has the potential for broader applications in dynamic environments such as hospitals. For instance, it could be adapted for real-time tracking of mobile medical equipment, such as ultrasound machines and crash carts, which are frequently moved across different rooms and require precise location tracking to ensure timely availability during critical situations. The adaptability and accuracy of this IPS position it as a central component in large-scale healthcare monitoring systems, offering substantial improvements in operational efficiency and resource management in healthcare settings. This opens avenues for future research and development focused on enhancing the system’s capabilities to handle complex, high-stakes environments where precision and reliability are paramount.
